# Pollination Biology and Life History Traits of the Rare Las Vegas Bear Poppy (*Arctomecon californica*)

**DOI:** 10.3390/plants13131762

**Published:** 2024-06-26

**Authors:** Sarit Chanprame, Terry L. Griswold, Joseph S. Wilson

**Affiliations:** 1 Department of Biology, Utah State University, Logan, UT 84322, USA; 2 U.S. Department of Agriculture, Agricultural Research Service (USDA-ARS), Pollinating Insects Research Unit, Logan, UT 84322, USA; 3 Department of Biology, Utah State University—Tooele, Tooele, UT 84074, USA

**Keywords:** Las Vegas bear poppy, pollination, conservation, bee declines, *Perdita meconis*, Mojave poppy bee

## Abstract

*Arctomecon californica*, the Las Vegas bear poppy, is a rare plant found only in the eastern Mojave Desert of North America. Because of recent declines in the populations of this endemic plant, conservationists are currently seeking protection for *A. californica* under the US Endangered Species Act. In this study, we investigated the natural history of *A. californica* and documented insect visitors potentially pollinating *A. californica* in Clark County, Nevada. We find that the populations of *A. californica* fluctuate from year to year, with many populations declining by over 90% from 2021 to 2022. The pollinator communities of *A. californica* also vary from year to year. In some years, specialist bees in the genus *Perdita* make up the majority of pollinators, while in other years, generalist bees like *Apis mellifera* and *Hylaeus* dominate. Furthermore, we confirm what previous work has suggested, that *A. californica* requires pollinators to set seed, yet not all insect visitors are good pollinators. This work provides useful natural history information about the Las Vegas bear poppy, which will be informative to conservationists designing strategies to protect this imperiled species.

## 1. Introduction

*Arctomecon californica* Torrey & Fremont, the Las Vegas bear poppy, is a rare perennial, herbaceous plant of the family Papaveraceae (Poppy family) [1], one of many plants endemic to the Mojave Desert. It is readily identifiable by its pale gray-green hairy, lobed leaves that are at the base of the plant and tall (0.3–0.5-m) branching stalks topped with deep yellow inflorescences [2,3]. *Arctomecon californica* is a gypsocline [4] primarily found on gypsiferous substrates, an environment which few plants can utilize. The result creates visual patches of yellow on the barren gray landscape during bloom. In this arid environment, such “islands” of resources may attract many visitors inhabiting the desert. Although *A. californica* does not produce nectar [5], it produces abundant pollen as a potential reward for visitors that feed on pollen (some beetles) or gather it for their young (bees).

*Arctomecon californica* is considered rare [6]. In 1993, the Bureau of Land Management conducted extensive surveys on *A. californica* in southern Nevada across 99 populations, with a combined estimate of 830,000 plants located mostly in Clark County, Nevada. This estimate likely represents a peak in population size due to the above-average precipitation in the area in the years preceding the 1993 survey [7]. Subsequent surveys showed *A. californica* in decline, and its range reduced to about half between 1993 and 2000 [8]. Many factors were suggested as causes of decline including habitat fragmentation due to human activities and urbanization, and changes in the pollinator community upon which *A. californica* relies [9]. Due to the reported declines in *A. californica* populations across its native range, a petition was made to the US Fish and Wildlife Service in July 2019 to list it as an endangered species under the Endangered Species Act [10].

Individual flowers of *A. californica* are short-lived, remaining open for approximately two days, at which point most, if not all, of the petals and anthers detach from the receptacle [9]. The stigmas of the flowers remain sticky for at least two days, allowing pollen grains to attach and fertilize the flower [9]. To improve the chance of fertilization and successful fruit set, *A. californica* continuously sends out short-lived flowers throughout an extensive season which starts as early as late February and continues into the beginning of June [9].

*Arctomecon californica* is reportedly reliant on bee visitors for pollination services as the plant is largely incapable of autogamy (self-pollination) or parthenogenesis (offspring without fertilization) [9], although a related species, *Arctomecon merriamii* Coville, exhibits different breeding strategies across populations (e.g., populations capable of autogamy) [11]. Diverse bees are known to visit *A. californica*, some more consistently than others. A survey conducted in Lake Mead National Recreational Area (LMNRA) in 1995 recorded 15 genera of Apoidea visiting the flowers [9]. They included both generalists (polyleges) and specialists (oligoleges, species that forage on only a few closely related species of host plants), natives and non-native bees. Two of these visitors, *Perdita meconis* Griswold and *Megandrena enceliae* (Cockerell), were noted as very important pollinators due to their abundance [9]. Both species were reported in a 2017 study [12]. While *M. enceliae* is viewed as an oligolege of creosote bush, *Larrea tridentata*, (DC.) Colville [13], *P. meconis* is an oligolege primarily on poppies in the genus *Arctomecon*, though they have also been collected on prickly poppies (*Argemone*) in some locations. In addition to these two abundant pollinators, *Perdita robustula* Timberlake was also reported, though in small numbers [9]. This bee is closely related to *P. meconis* and is broadly associated with poppies but has a wider distribution in the xeric Southwest [14]. These pollinators, in the aggregate, are thought to be the limiting factor in fruit and seed set; researchers found that flowers that were cross-pollinated experimentally by hand resulted in greater seed set [9].

It is noteworthy that not all visitors to *A. californica* are good pollinators. The combination of synchronous dehiscence of anthers of *A. californica* and the very open structure of the flowers allows diverse insects to visit and take pollen from the flowers. Many small bees are thought to be “gleaners” due to their size and behavior, collecting pollen grains without ever touching the stigma, and therefore, not pollinating the plant. In contrast, larger bees, due to their size, cannot avoid contact with the stigma and deposit pollen grains in the process. This, however, does not mean that bigger pollinators are always better pollinators. Because of their size, they have greater pollen-carrying capacity, which for flowers with no rate-limiting mechanisms means that pollen may be harvested in a few trips, quickly depleting the anthers. The fidelity of visitors also affects the success rate of fertilization and fruit set as generalist pollinators may deposit incompatible pollen grains, thereby clogging the stigma [15]. In this sense, not all visitors to *A. californica* are pollinators, and not all pollinators are equal.

From the early description of *A. californica* in 1845 to the peak bloom surveyed in 1993, Clark County has undergone two major changes. One is climate, particularly changes in the amount and timing of precipitation. Weather data from the weather station at McCarran Airport in Las Vegas document decreasing precipitation and shifting precipitation patterns, with 2020 reporting 240 days of no measurable precipitation, breaking the previous record set in 1959. The other major change is the rapid growth of the human footprint in the county and particularly greater Las Vegas. Based on our observations, some populations of *A. californica* that were thriving in early studies of the species [9] have been extirpated, as the habitat where they lived is now covered in housing subdivisions.

Climate patterns likely have direct effects on the plant itself. The effect of climate change has been observed in the phenology and productivity of some plants [16]. While the effect of climate pattern disturbance is harder to assess in rare, specialist species like *A. californica*, it is crucial to learn more about the plant’s natural history to help guide future conservation efforts. Indirect negative effects on *A. californica* are also expected via impacts on the pollinator communities. Various studies have predicted the effects of climate change on pollinator communities with various degrees of positive and negative impacts reported. These studies can differ greatly in their projections. For instance, a study about energetics suggests that with warming climate, species richness in a given area will increase [17], while another study [18] predicts declines of pollinators due to changes in their environment that exceed their ability to adapt. What these studies do agree on is that climate change will likely drive changes in pollinator communities.

With shifts in pollinator community composition can come changes in productivity for plants which rely on their pollination services. For a species such as *A. californica*, where pollination is limiting [9], changes in pollinator composition are more likely to affect productivity and ultimately the survival of any given population compared to plant species not reliant on pollinators.

The direct effect of climate change on the plant, combined with the changing pollinator community which *A. californica* is reliant upon, might put even greater pressure on *A. californica*’s already limited and apparently declining populations. Our objectives are to: (1) Expand understanding of the phenology and floral behavior of *A. californica*; (2) Survey and document floral visitors to *A. californica* in Clark County; and (3) Determine the efficacy of common Apoidea visitors and non-Hymenoptera to *A. californica* as pollinators.

## 2. Results

### 2.1. Blooming Season

**Figure 1:**
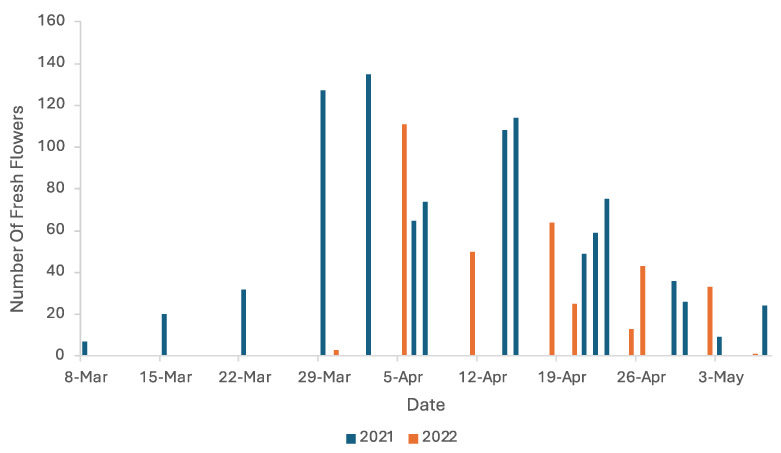
Number of freshly opened flowers observed on 10 marked, flowering plants from across the study sites throughout the blooming seasons of 2021 and 2022.

Blooming season of *A. californica* in 2021 in Las Vegas/Lake Mead National Recreation Area began around mid-March and concluded in late May, with the first flowering plant observed at the Pb site on 8 March and the last flowering plants observed at PC on 7 May (Figure 1), the last day of observation due to declining activity of visitors to near zero. It is important to note that although it was the last day of observation, many larger plants on Gold Butte National Monument (PC, RBS) and on the LMNRA side (RGN, RR) still had fresh buds in development, which would potentially push the end of flowering season to mid- to late May (14–20s) as new buds were observed during the surveys to take anywhere from 6 to 10 (and most often 7 to 8) days.

In 2022, the blooming season of *A. californica* in the same area began in late March and concluded in early May, with the first observation of a flowering plant at the BW site on 30 March with no sign of older flowers, and the last flowering plants observed at site Pb on 6 May, with the last four flowering plants on site with no new buds present (Figure 1). The end of bloom can be abrupt. At the farthest east population of *A. californica*, Vulture Ridge in western Grand Canyon National Park, visited on 12 May, of 243 reproductive plants, 168 were in fruit only, 72 had at least one first-day flower, and only 3 had buds (T. Griswold, personal observation). Our observations indicate that late spring rains do not appear to engender additional budding. For example, a burst of precipitation observed on 22 April in the LMNRA area covering sites SP, BS1, BS2, BS3, and BS4 did not facilitate more bloom or new bud generation.

### 2.2. Population’s Fluctuation in Arctomecon californica

Extreme changes in *A. californica* population size occurred between successive years (Table 1). Sites surveyed in both 2021 and 2022 showed fluctuation in the number of living plants and number that bloomed. Many poppy populations experienced over 80% loss from one year to the next. Most of the plant losses at several sites were seedlings, though a few bigger plants were also lost due to damage by vehicles. At some sites, there was less loss from one year to the next, but there was a decrease in the number of blooming plants between years. It is important to note that percentage values (Table 1) can be misleading in some cases since the small total number of plants at small populations exaggerate the percent change.

### 2.3. Insect Visitor Survey and Behavior

**Figure 2:**
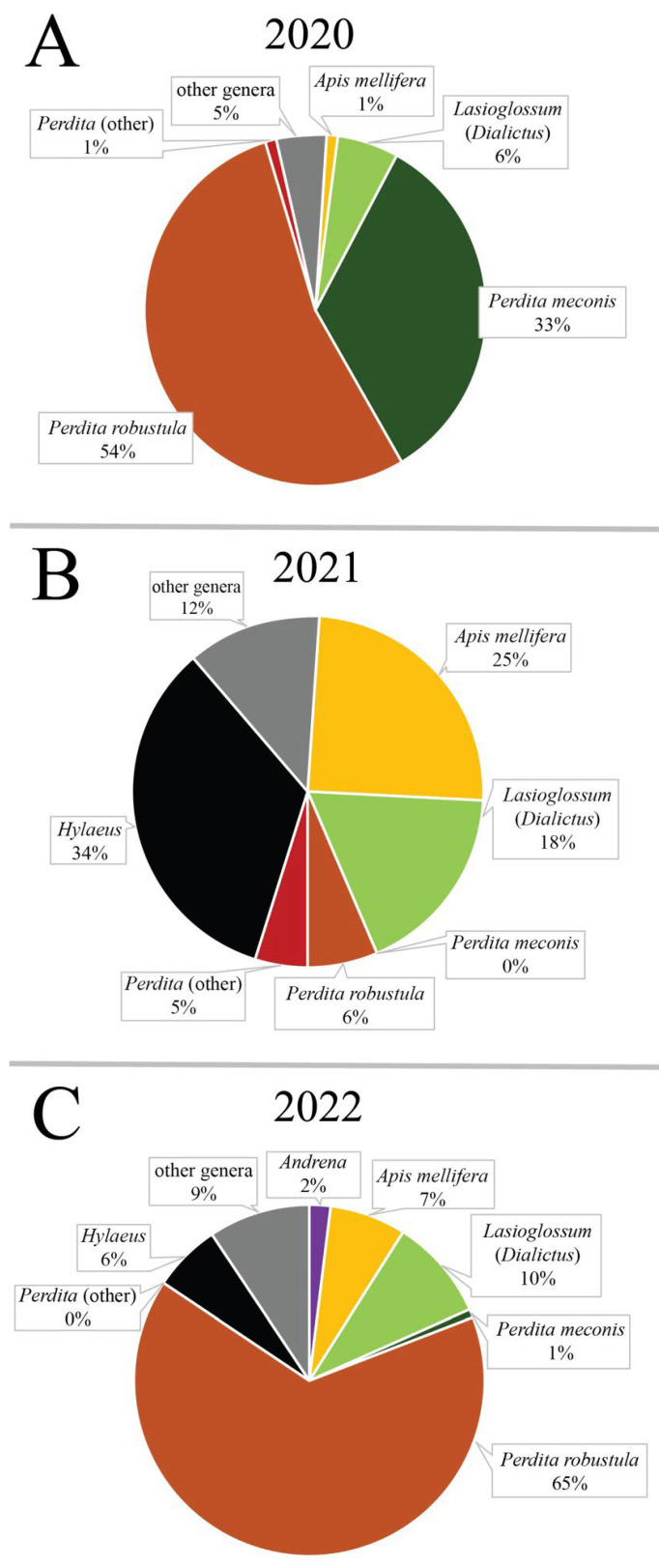
Bee communities associated with *A. californica* in Clark County Nevada, 2020–2022. (**A**). shows the ratio of different bees collected in 2020, (**B**). shows the he ratio of different bees collected in 2021, and (**C**). shows the ratio of different bees collected in 2022.

A total of 22 different bee genera were detected visiting *A. californica* in Clark County from 2020 to 2022 (Table 2). In 2020, eight genera of bees were collected from *A. californica*, with *Perdita* being the most abundant and widespread (Figure 2, Table 2). *Perdita robustula* was found at every site surveyed in 2020 except BW. *Perdita meconis*, however, was only found at RGN, BS1, and RS. Though not as widely distributed as related *Perdita* species, it was abundant when present. The visitor community of *A. californica* in GBNM was more diverse with seven genera detected compared to four genera in LMNRA. Three genera of bees (*Andrena*, *Ashmeadiella*, and *Perdita*) were found in both areas. 

In 2021, 12 genera of bees were detected from *A. californica* (Table 2). Bees from the genus *Hylaeus* were the most abundant, accounting for 34% of all visitors. *Apis mellifera* L. was the second most abundant, accounting for approximately 25% of all visitors (Figure 2). Their numbers were not as high as *Hylaeus*, but they were more consistently present as they were detected at every site except OCM and BW (which never yielded any visitor observations that year). Bees from sweat bee genus *Lasioglossum* (the vast majority in subgenus *Dialictus*) accounted for 18% of all visitors and were also detected consistently, present at nine sites. *Perdita robustula*, which was the most abundant in 2020, dropped from 54% to 7% of all visitors, and *P. meconis* was never detected in 2021. Increased diversity of visitors was found in LMNRA with 10 genera of bees compared to 9 genera detected in GBNM area, with six genera of bees (*Andrena*, *Apis*, *Colletes*, *Hylaeus*, *Lasioglossum*, and *Perdita*) detected in both areas.

In 2022, 16 genera of bees were detected from *A. californica* (Table 2). Bees from the genus *Perdita* were the most abundant, accounting for approximately 66% of all visitors (Figure 2). Of this total, the vast majority were found to be *P. robustula*, with only two individuals identified as *P. meconis*. Bees from *Lasioglossum* were the second most abundant, accounting for approximately 9%. Bees from genera *Hylaeus* and *Apis mellifera*, which were detected most frequently in 2021 (34% and 25% respectively), accounted for just 6% and 7%, respectively, in 2022. The community of visitors associated with *A. californica* was more diverse in GBNM, with 14 genera detected, while 8 genera were detected in LMNRA, with 6 genera of bees in common (*Andrena*, *Anthophora*, *Eucera*, *Hylaeus*, *Lasiglossum*, *Perdita*).

### 2.4. Pollinator Efficacy

**Figure 3:**
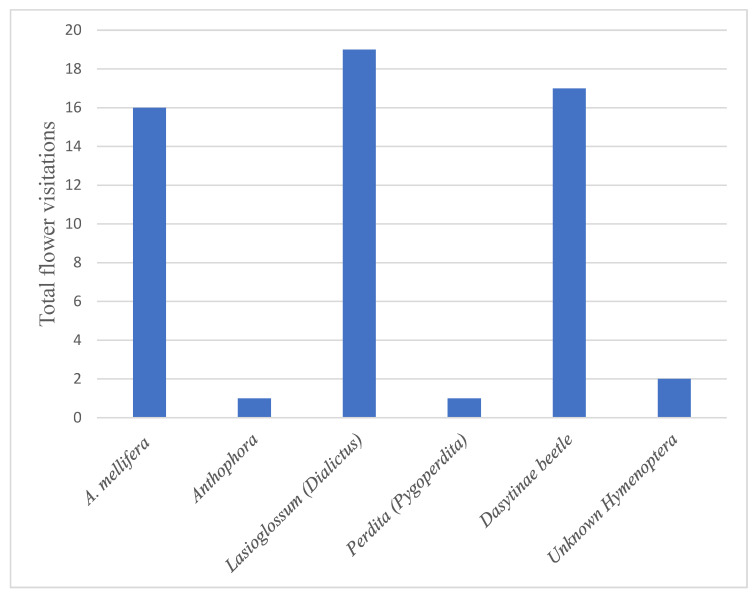
Total number of flower visits by the six different floral visitors observed in the pollinator efficacy study.

The pollinator efficacy experiment was conducted as part of the 2021 pollinator surveys. While there were many types of visitors collected that were associated with *A. californica*, the observers were only able to observe four bee species visiting the flower during the timed trials (*A. mellifera*, *Anthophora*, *Lasioglossum* subgenus *Dialictus*, and *Perdita* subgenus *Pygoperdita*) and one coleopteran (Dasytinae). The majority of floral visitation observations were of *A. mellifera* (*N* = 16), *Lasioglossum* subgenus *Dialictus* (*N* = 19), and dasytine beetles (*N* = 17), with two visits from unknown Hymenoptera and only a single individual observation each of *Anthophora* and *Perdita* (Figure 3).

#### Average Fruit and Seed Set

Total visitation (Figure 3) was not necessarily connected to successful fruit set. Fruits developed from flowers visited by *A. mellifera*, dasytine beetles, *Perdita* (*Pygoperdita*), and from unknown Hymenoptera. Visits from *Lasioglossum* (*Dialictus*) did not result in any fruits being produced. In contrast, the single flower visited by *Perdita* (*Pygoperdita*) successfully set fruit and survived to fruit maturity, despite the similar sizes of *Perdita* and *Lasioglossum*. The control flowers that never had their bags removed all failed to set any fruit, and the control flowers open to pollinators all successfully set fruit.

**Figure 4:**
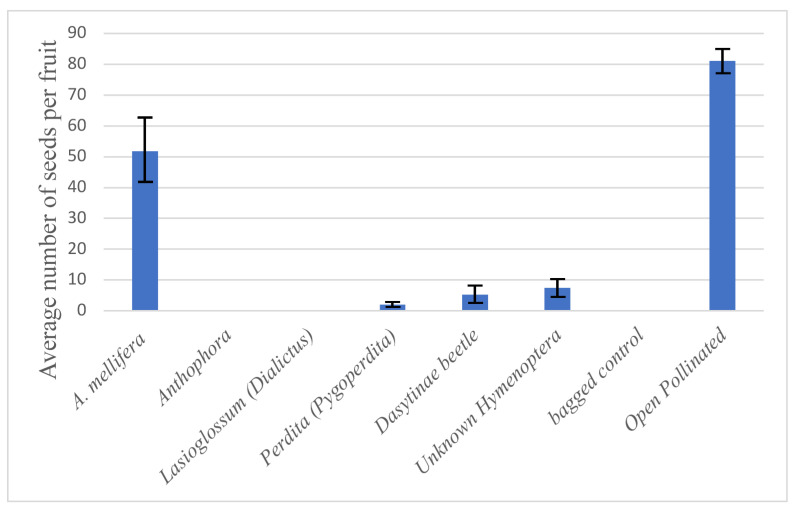
Average seed count with standard deviation for flowers visited by the six different floral visitors observed in our study.

The majority of seeds sets from flowers were produced by flowers visited by *A. mellifera* (Figure 4). Although the fruits were harvested before they were fully mature, the size differences between aborted seeds and developing seeds were distinct enough to tell them apart. Flowers visited by dasytine beetles and *Perdita* (*Pygoperdita*) also successfully yielded seeds, although in very small numbers. The flower briefly visited by *Perdita* (*Pygoperdita*) only yielded two seeds. The control flowers open to all pollinators successfully set seeds with the average open-pollinated flower producing about 60% more seeds than flowers visited by *A. mellifera* and over 3900% more seeds than the single visit by the *Perdita* (*Pygoperdita*). It should be noted that the controls potentially received multiple visitors. The floral visits by *Anthophora* and *Lasioglossum* (*Dialictus*) yielded no seeds.

### 2.5. Visitor Behavioral Observations

**Figure 5:**
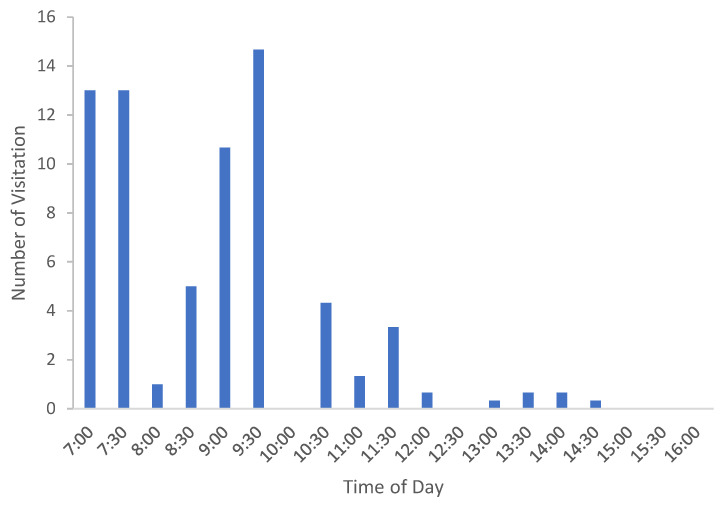
Average visitation of Apoidea to *A. californica* in relation to time of day (2021).

**Figure 6:**
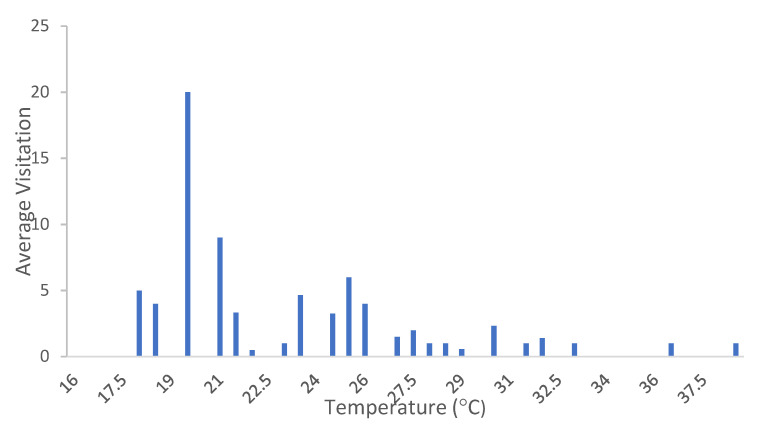
Average visitation of Apoidea to *A. californica* in relation to temperature (2021).

Most insect visitations were recorded early in the day including the *Perdita*, with records rare in the afternoon (Figure 5). Ambient temperature appears predictive in relation to visitation rate. Visitation rate (based on 15 min timed observations, see Section 4.3.4) increased steadily from the first measurement around 07:00, with ambient temperature of approximately 18 °C (Figure 5 and Figure 6). The peak of visitor activity was approximately 09:30, when ambient temperature measured approximately 20 °C. As the temperature increased to 27 °C, the visitation rate declined sharply.

Observations of behavioral patterns for specific visitors provide insight into their effectiveness as pollinators of *A. californica*. Because these were observations of live subjects, some visitors were identified only to genus or subgenus level, as further identification could not be accurately carried out without collection. Many of these observations were made opportunistically and not part of the timed pollinator efficacy study. Most of these observations are preliminary, and further study must be conducted to determine if the patterns we noticed happen consistently.

*Apis mellifera* (honey bees). During the 2021 bloom, the earliest visitors, *A. mellifera*, arrived about half an hour after sunrise and all but ceased by mid-day, with sparse visitation in the afternoon. Upon locating a desired flower, they would land on a part of the flower which granted them the easiest access to the anthers, usually the petals. They would gather pollen from the anthers using their fore legs, pass the pollen grains along to their mid-legs, and then pack the pollen onto their corbiculae. They often walked over the stigma to access anthers located on the opposite side of the flower from which they landed. Once they finished with one flower, they would typically make short flights to visit multiple flowers on the same plant, and then seek out a nearby flowering plant to continue foraging. From the pollinator efficacy experiment, *A. mellifera* was found to be the only visitor of *A. californica* that consistently induced seed set (Figure 4). Their foraging habits of walking back and forth over the flower as they gathered pollen and their relatively large size, combined with their abundance, likely contributed to the successful seed sets of *A. californica*.

*Anthophora.* The *Anthophora* visitors to *A. californica* were slightly larger than *A. mellifera*. They became active later in the morning as the air warmed up. *Anthophora* are fast flying and rarely landed on the flower. When they landed, they spent only a brief moment, no longer than two seconds, gathering pollen grains from the anthers, and then flew off, presumably to nearby flowering plants. Whether they touched the stigma of the flower or not was largely determined by where they initially landed, as they did not exhibit the same meandering foraging pattern on the flowers exhibited by *A. mellifera*. They rarely visited multiple flowers of the same plant, which in theory should promote outcrossing of the plant and result in high visitor efficacy; however, more data are needed to determine their effectiveness.

*Hylaeus. Hylaeus* visitors of *A. californica* are small (body length 5–7 mm), mostly hairless bees. They were present at most sites and became active in the morning about two hours after sunrise. We observed *Hylaeus* landing on the petals, anthers, and stigma with no particular preference. They made multiple contacts with the stigma during their foraging bouts. Their visits to an *A. californica* plant were usually brief, lasting about 10–15 s, with the longest recorded at 42 s. Once they were done, they walked away from the anthers and slipped out of the flowers in the gaps between petals. They usually visited multiple flowers on the same plant. Their presence was observed throughout the day, from morning until late into the afternoon. Their foraging activities ceased around mid-day, however, when temperatures reached 32 degrees Celsius. At that point, they took shelter from the heat inside the flower, in the crevasses between the petals and the anthers. Despite their wide distribution and relative abundance, they are unlikely to be an effective pollinator as pollen grains do not readily stick to their hairless body. Furthermore, *Hylaeus* carry pollen internally so very little, if any, remains stuck to the outside of their bodies. During the efficacy experiment, no *Hylaeus* ever made contact with any of the marked flowers, making it difficult to ascertain their potential efficacy.

*Lasioglossum* (*Dialictus*). *Dialictus* are minute visitors (4–6 mm in body length) to *A. californica*. They were ubiquitous across sites and fairly abundant. Their visitation started early in the morning alongside *A. mellifera*. They preferred to land on the petals and access anthers from underneath. It appeared that they were too small to pull anthers towards them, so they were mostly seen hanging onto the anther while foraging and packing pollen balls onto their hind legs. Once they finished collecting pollen, they would take off from that position, which usually was atop the anthers. Small amounts of pollen could possibly be flung onto the stigma during their take off, although none of the flowers they visited during the experiment successfully set seeds.

*Perdita robustula.* While *P. meconis* was never found in 2021, the closely related species, *P. robustula*, was found at one of the sites active early in the day. They were minute in size, similar to *Dialictus*, but their foraging behaviors differed significantly. While mostly preferring petals as their landing site like *Dialictus*, *P. robustula* males consistently walked all over the flowers, including over the stigma. *Perdita robustula* females engaged in mating while foraging, dragging their partners through the anthers and covering both of them in pollen. When the female was done foraging at a particular flower, she would seek out a takeoff site where she could be parallel to the ground, which on many occasions was the stigma. We noticed many *P. robustula* females carrying pollen balls already on their hind legs when they landed, indicating that they visited multiple flowers during a foraging trip. Although not the same species, the behavioral pattern of *P. robustula* seems closely aligned with that of *P. meconis*. As was hinted at in the *P. meconis* study [9], and supported by later reports [12], this could make them very effective pollinators as a species or species complex. However, this was not the case in 2021 as they lacked the abundance to make them principal pollinators. During our efficacy experiment, only one flower was visited by *P. robustula*. While seed set was induced in that particular flower, the seed yield was very small, further emphasizing the relationship between their abundance and effectiveness.

*Megandrena enceliae. Megandrena enceliae* is a species of mining bees whose range is limited to the southwestern deserts of the United States. They have complex relationships with their narrow range of host plants. Male *M. enceliae* appear to be less selective in their floral preference, while females are more selective, mostly observed and collected on *A. californica*. They are 13 mm in body length, considerably larger than most other visitors to *A. californica*. In the past, they were reported as one of the most important visitors of *A. californica* in Clark County [9,12]. *Megandrena enceliae* was only detected at *A. californica* in 2022.

Cleridae and Dasytinae. Clerid and dasytine beetles were a common sight on *A. californica* flowers at almost all sites. They were inside the flower from early morning to late at night, as they seemed to sleep inside the flower. They foraged inside the flower, with some of the clerids and dasytines foraging for pollen grains, while other clerids preyed on other beetles and bees visiting the flowers. Although they were fully covered in pollen on many occasions, they may not have made good pollinators for *A. californica* as they rarely moved from one flower to another, and even more rarely left the plant for another flowering plant. Data from our efficacy study in 2021 indicated that they occurred in relatively high abundance, and made contact with flowers fairly often, but those flowers only produced minimal seeds.

### 2.6. Floral Life History and Phenology

*Arctomecon californica*’s reproductive season starts each spring with many reproductively mature plants in each population sending out flower buds on stalks which elevate the buds to the height of 13 to 55 cm from the ground. Flower buds are initially pendant. The flower buds increase in size as they develop, sepals remaining pale green until one or two days before the opening of the flower, at which point the sepals gradually turn yellow, the bud becomes erect and points upward. The sepals wither and detach from the flowers when the flowers are fully open. Flower buds take roughly one week to develop into a mature flower. New flower buds can emerge from matured stalks continuously throughout the season as the main stalk branches off to support more flowers. Flowers begin to open as early as 01:30 and take about one hour to fully open. Many flowers on each plant can bloom in a single night in a staggered manner from 01:30 to 04:00, at which time all flowers that will bloom that day are fully open and all anthers have dehisced.

Larger plants bloom earlier compared to smaller ones and send out more flowers over a longer blooming season. The smallest observed blooming plant was located at OCM in 2021. The plant was 10 cm in diameter and only produced a single flower throughout the 2021 season; it did not bloom in 2022. The largest observed blooming plant was located at Pb in 2021. The plant was roughly 50 cm in diameter and sent out more than 200 flowers during the 2021 season, and a comparable number during 2022.

After the flower opened and all the anthers had dehisced, the first visitors arrived. At the nine locations we observed in 2021, most of the pollen available had been harvested by insect visitors before 12:00 of the first day, and empty anthers started to wither and easily detach from receptacles by the end of the day. Petals of flowers remained firmly attached to the receptacle on the first day and most of the second day, although the color of the petals became less vibrant. Petals easily detached from the receptacle by the third day of bloom.

Despite the early opening of the flowers before sunrise, pollination by moths is unlikely as moths were only rarely observed visiting the flowers. In one of the observation bouts, a single moth was observed to visit a nearby flower of *A. californica*. The moth landed on the outside of the flower and did not try to get inside. Upon further examination, the flower was found to be a second-day flower with detached anthers. The moth, in this instance, was likely attracted to the flower by the observer’s headlamp. Inspection of flowers pre-dawn showed clean stigmas with no sign of pollen deposition.

In 2022, damage to plants was also observed to varying degrees at multiple sites. Insect damage was more prominent compared to 2021 observations. At HH, large numbers of grasshoppers were eating flower buds and fruits throughout the season. Herbivory damage from large mammals was also observed at multiple sites, with PC receiving the most herbivory damage. At PC, a particularly large *A. californica* was observed uprooted, missing most of the plant, with only a few basal leaves remaining.

## 3. Discussion

### 3.1. Blooming Season

The blooming season for *A. californica* varies significantly even between successive years. In 2021, bloom lasted approximate 60 days (early March to mid- to late May), considerably longer than the approximately 40 days in 2022 in the same area with the first flower recorded in late March. But neither approaches the length of bloom in 1995, which lasted approximately 80 days [9]. The current shorter blooming season could be related to the drought cycle the region has experienced over the last few years. The previous study [9] also indicated that two populations of *A. californica* that experienced a pulse of precipitation responded with a secondary bloom peak. The similar precipitation event in 2022 did not result in any secondary peak of bloom, nor increased bloom, despite happening at about the same date as the one reported in 1995, although in comparison with the corresponding blooming season, it was much later, perhaps too late in the phenology to have any effect. It could also be that the quantity of precipitation was too low to have an impact. The precipitation event in 1995 provided 0.75 cm over a period of a few days, while the precipitation recorded in 2022 was about 0.17 cm and only lasted for about two hours. This shorter blooming season could affect the productivity of the plants as the population could produce fewer fruits and seeds during a shorter reproductive period. It could also lower productivity if the plant’s reproductive period became misaligned with native pollinator phenologies. The effects of a shifting seasonality on *A. californica* propagation and persistence should be studied more to further our understanding of their relationship and improve the effectiveness of future conservation methods.

### 3.2. Fluctuation and Decline

The significant interannual fluctuations of *A. californica* populations described in the past are mirrored by that observed between 2021 and 2022, especially in study sites in the LMNRA group. One of the reasons proposed to explain this fluctuation is the culmination of *A. californica*’s short lifespan combined with unpredictable seedling establishment due to erratic conditions [4]. This reason alone cannot explain the sudden mass die-off observed in many populations over the last couple of years. The recent losses were not limited to larger, older plants but extended to small, and minute (seedlings) as well. This sudden loss across all demographics cannot be explained by a large cohort of plants reaching the end of their natural lifespan. Although the greatest losses were observed at sites in close proximity to each other (RGN, RGS, and SR), other sites in LMNRA such as Pb, Apex, and RR also exhibited losses, although not to the same extent. Our observations indicate that most of the plant losses in these sites were seedlings and smaller plants, while the larger, well-established plants at each site were alive with varying degrees of healthiness. This loss of seedlings and smaller plants might indicate harsh conditions that only the well-established plants can survive. The weather data reported from McCarran International Airport support this interpretation. They show a trend of lower, more sparse precipitation in the area since the early 1990s, alongside a temporal shift of peak precipitation to later in the year. It is possible that these changes in weather patterns, alongside the natural short lifespan of *A. californica*, account for the losses that we observed during our study period.

### 3.3. Visitor Surveys

The comparison of visitor communities around *A. californica* from 2020 to 2022 revealed a drastically shifting nature of visitor types from year to year even within the same localized area. The percentage of *Perdita*, an important visitor to *A. californica* according to previous reports [9,12], shifted from 84% in 2020 to 11% in 2021, to 66% in 2022. The same changes were also observed in other genera of bees to varying degrees.

A previous report [9] documented similar generic diversity with 15 bee genera visiting *A. californica* flowers in the LMNRA; nine of these overlap with the current results. Although the richness at genus level stayed similar, the turnover across decades and between consecutive years suggests that many of these genera likely do not share the loyalty to *A. californica* that *P. meconis* and *P. robustula* exhibit, and thus do not provide predictable pollination services. The combined data also highlight the spatial component of the shifting visitor communities over the 25-year period, as most of the sites observed in 1998 no longer have poppy populations, although the sites selected for the 2020–2023 surveys were within the same general area (LMNRA). RGS and SR are within 500 m of one another, and yet visitors of *A. californica* were not identical. 

It is also noteworthy that while a specific genus of bees can be detected at a specific site, this does not equate to consistent presence across time. *Hylaeus* was the most abundant in 2021, detected at 10 sites ranging from the westernmost site in LMNRA to the easternmost site in GBNM, but not every observation/collection event at those sites detected them. Similar gaps in detection are expected for other bees to varying degrees. This suggests more nuance on bee behaviors and environmental conditions, which promotes different species in a given area to begin and sustain their activities. These gaps in bee detection along with year-to-year fluctuations are likely to pose further challenges for any attempts to make comprehensive visitor surveys and catalogue particular environments in the future. 

### 3.4. Pollinator Efficacy

Although the pollinator efficacy study suggests trends about potential contribution from different types of visitors, it is definitely not conclusive; first, because common visitors like *Hylaeus* never made an appearance during the pollinator efficacy study, and species that were common the previous year (e.g., *Perdita meconis*) were absent.

Our findings corroborate the previous study of the *A. californica* reproductive system [9], indicating that *A. californica* is largely self-incompatible and requires pollinators for reproduction. It is also clear from the results that *A. californica* benefited from repeated visitation from various types of visitors. The bagged fruits with limited exposure to visitors yielded fewer seeds per fruit on average in comparison to fruits which did not have their visitor types and exposure time restricted.

Our data clearly show that of all the observed flower visitors, honey bees, *Apis mellifera*, were the most effective pollinator of *A. californica*. Other visits that resulted in successful seed set included visits by unknown Hymenoptera, Dasytinae beetles, and *Perdita,* though these visits yielded far fewer seeds than the honey bee visits (Figure 4). This indicates that honey bees, though not native to the Mojave Desert, might be playing an important role as pollinators of *A. californica*, especially when native bees like *Perdita* are absent or present in few numbers.

In our study, the open-pollinated controls yielded many more seeds than any of the experimental flowers that were visited by pollinators. This could indicate that other pollinators not observed in our experiment contributed to the open-pollinated plants. Or, more likely, this suggests that a single visit by any one pollinator (like the visits observed in our study) are not enough to fully pollinate the flower. In other words, *A. californica* flowers produce more seeds when visited multiple times or by multiple pollinators.

Diurnal activity patterns of early maximum activity decaying across the morning and near absent in the afternoon were consistent, but the cause(s) are unclear. It appears that there is an ambient temperature threshold of approximately 18 °C, at which point visitation rises rapidly from zero to peak activity at approximately 20 °C. From then on, visitor activity decreases until activity nearly ceases at approximately 27 °C. Whether this is a reflection of bee physiology, behavior, or decreasing availability of pollen is unclear. The drop coincides with observed withering of anthers in first-day flowers with presumed depletion of pollen. From these observations, it is likely that, while there is an ambient temperature threshold which needs to be crossed before activity can begin, the level of activity around *A. californica* is strongly tied to availability of fresh anthers and pollen rather than the ambient temperature.

The sample size of this study is small, especially compared to many experiments of a similar nature. Additional work is needed that balances the impact of the study on the rarity of *A. californica* and weighs the impact of repeated visits across this fragile ecosystem with gaining further insight into pollinator function.

### 3.5. Floral Life, Plant Phenology, and Interaction with Visitors

Elements of our observations of *A. californica* life history suggest some differences compared to that reported in the 1990s [9]. For example, the longevity of individual flowers is less than what is inferred from the earlier study. This could potentially be related to a period of greater precipitation in the early 1990s in contrast to the recent drought. It coincided with the reported peak bloom in 1993 [7], from which point the *A. californica* population has steadily declined. The observed differences may stem from the plant’s response to lower precipitation rather than evolving adaptation, as the shortened lifespan of flowers was observed at every site.

## 4. Materials and Methods

### 4.1. Study Sites

**Figure 7:**
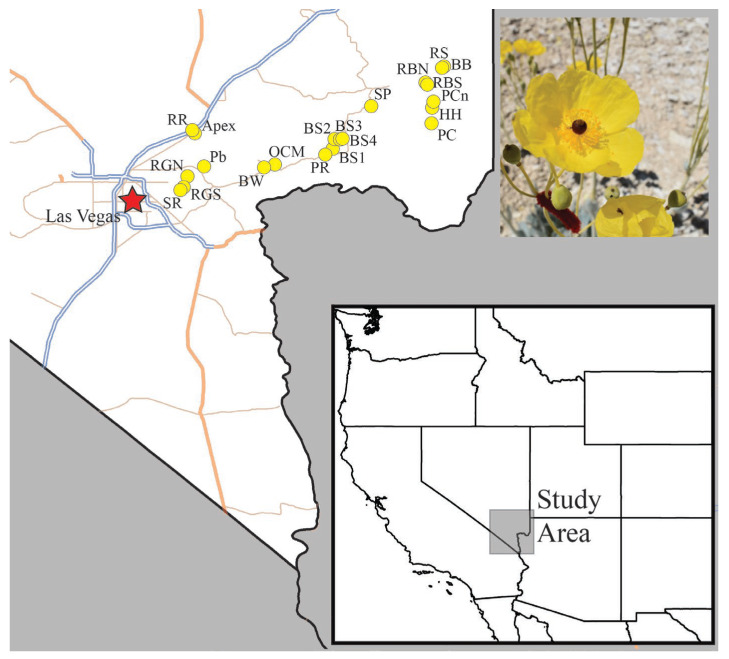
Map showing the study area and individual study sites surveyed in this study. Site names correspond to Table 2.

The association of *A. californica* with substrates with high concentrations of gypsum is well documented. Study sites were selected based on historic *A. californica* populations [9,12]. These sites were then surveyed on the first week of each field season to determine usability based on number of potentially flowering plants, density of the plant population, and historic collection records of *P. meconis*. Details for locations studied in one or more years (2020–2022) are found in Table 3 and Figure 7.

### 4.2. Floral Life History and Phenology

Exploration of flowering behavior was conducted in 2021 and again in 2022. Initial fieldwork found flowers were open early in the morning, but the timing of anthesis was unknown. We selected site RBS due to the abundance and size of flowering plants, ease of access to those plants, and the minimal amount of low ground-covering debris and vegetation. Two individual plants were marked using plastic flags with different markings on them to ensure that the observers could locate and identify the same plants every time even in low or unfavorable light. Four flower buds from each plant were marked using different color twist ties. From 18:00 of Day 1 to 12:00 of Day 2, a photo of each flower bud was taken every 30 min to observe their progression over time. From 13:00 of Day 2 to 7:00 of Day 3, the frequency of photos taken was reduced to one photo every hour per flower. Observers also took notes of floral changes and events that occurred during an observation bout.

To document plant phenology, the number of living plants and flowering plants (recorded mid-April, nominally middle of recorded blooming season) were counted from sites we extensively surveyed. Blooming season length was estimated by measuring the time between the first flower observed and last flower observed or estimated. The last flower estimate was made using data collected from the observation of the floral life portion of the study and observations made throughout the season. The collected phenological data from 2021 and 2022 were then compared to one another and to historic bloom phenology [9].

### 4.3. Pollinator Sampling

#### 4.3.1. Pollinator Visitor Surveys

In 2021, pollinator surveys were conducted at ten sites: Apex, RR, BS2, RGN, RGS, SR, PC, RBS, RS. All surveys consisted of two components, visual observations and net collections, conducted by a pair of field technicians who alternated the two methods simultaneously for two rounds (see details below). This was carried out to expand the temporal and spatial coverage of each method and decrease the chance that some pollinators evaded the survey due to their low abundance or phenological mismatch.

#### 4.3.2. Visual Observation

Visual observations consisted of five flowering *A. californica* plants spread across the population selected by each observer, thus totaling 10 per site. Environmental data (site name, date, time, weather condition, wind speed, and temperature) were recorded at the initiation of each observation period. Upon arrival at an individual plant, the observer positioned themselves approximately one meter away from the plant, putting the flowering plant between themselves and the sun so as not to cast a shadow on the plant. For five minutes, researchers watched the plant from their position, pollinators visiting each plant were recorded to genus level and, when possible, species, with minimal interference with the pollinator’s activity. Plant specific data such as number of flowers, buds, and fruits were also recorded. In addition to the timed visual observations, opportunistic observations were also made while researchers visited each site. These opportunistic observations allowed us to observe and record behaviors from pollinators not present during the timed observations.

#### 4.3.3. Net Collection

At initiation of net collections, date, time, temperature, and wind speed were recorded. The collector visited 50 flowering *A. californica* plants, or if fewer plants were in bloom, the total number visited was recorded. Pollinators present at arrival to each plant were collected with special care not to damage the flowers.

#### 4.3.4. Visitation Rates

Visitation rates were measured by counting Apoidea visitors making contact with flowers during the pollinator efficacy experiment. Each contact counted toward the rate of visitation, even if it was the same individual making repeated contacts, as it is difficult to distinguish individual visitors when they are in flight.

### 4.4. Pollinator Efficacy

Pollinator efficacy studies were conducted in 2021 at three sites chosen because they had the strongest populations of potentially flowering *A. californica* plants: RGN (located east of Las Vegas), and PC and RBS (located within Gold Butte National Monument). Optimally, the number of sites and number of bagged plants would have been larger but was restricted due to concerns of putting pressure on the already apparently declining populations of both *A. californica* and its associated rare visitor *P. meconis*.

The experiment was conducted by a two-person team at each site. On the evening of the previous day, 20 flowering plants were selected at random at a site across the population. Medium- to large-size flowering plants were preferred due to the higher chance of obtaining open flowers for the observation and stronger flower stalks that reduced loss of seed pods due to winds. One inflorescence with mature buds was selected from each plant and covered with a large fine mesh bag that encompassed the entire inflorescence and was tied closed with twist ties to prevent access to the flowers by insects. On the day of observation, each observer positioned themselves at least a meter from a selected plant and removed the mesh bag from the inflorescence.

Two first-day flowers were observed simultaneously for insect visitors for up to 15 min, recording the identity of any visitor along with time, temperature, and the number of instances it or other individuals of the same species made contact with each flower. The observation concluded when the first type of visitor left the plant, or the allotted time of 15 min had been spent. In some instances when more than two freshly bloomed flowers were present inside the large mesh bag, one flower was bagged immediately to completely exclude visitors. The two flowers were then covered with smaller mesh bags and closed with color-coded twist ties to indicate interaction (type of visitor, or the lack thereof) and labeled with a unique flower ID. There were two instances where an unidentified Hymenoptera briefly visited the flower but flew away before it could be identified; these are included in the analysis as “unknown Hymenoptera”. At the end of the season when fruits had matured, the fruits were collected, opened, and their seeds counted. In addition, 20 mature fruits from unbagged open-pollination plants flowering at the same time were collected from each site, and their seeds were counted to provide baseline productivity of each site. Once the data had been recorded, all seeds were returned to their sites of origin so as not to remove seeds from the seedbanks of the various populations of this imperiled plant.

## 5. Conclusions

Our research into the pollination biology of the rare plant *A. californica* confirms that it requires pollinators in order to make seeds. Furthermore, we found that the pollinator community can shift wildly from year to year, with some years dominated by native pollinators, and others dominated by honey bees. Our pollination efficacy studies and pollinator observations suggest that some native bees like *Lasioglossum* are likely poor pollinators, and some native bees like *Perdita* can be good pollinators. Also, we find that the non-native honey bees (*Apis mellifera*) can be good pollinators of this rare plant. Our research provides some baseline data and useful natural history that conservationists and land managers can use to make more informed decisions about how to best protect the dwindling populations of *A. californica*, the Las Vegas bear poppy.

## Figures and Tables

**Table 1:** Comparison of living and blooming *A. californica* from overlapping surveyed sites between 2021 and 2022.

Site	Code	2021		2022		Changes in Living Plants	Changes in Blooms
		Alive	Bloom	Alive	Bloom		
**Lake Mead Area**							
Apex	Apex	68	22	12	12	−82%	−45%
Railroad	RR	52	25	37	17	−28%	−32%
Bitter Spring 2	BS2	145	35	138	28	−4%	−20%
Rainbow Garden North	RNG	153	59	4	0	−97%	−100%
Rainbow Garden South	RGS	96	47	3	0	−96%	−100%
Shooting Range	SR	82	33	4	0	−95%	−100%
Pabco	Pb	149	52	108	27	−27%	−48%
**Gold Butte Area**							
Helicopter Hill	HH	74	29	68	32	−8%	+10%
Poppy City	PC	259	67	236	63	−8%	−5%
Red Bluff North	RBN	294	20	315	26	+7%	+30%
Red Bluff South	RBS	196	35	183	44	−6%	+25%
Restoration Site	RS	255	1	270	4	+5%	+300%

**Table 2:** Apoidea visiting *A. californica* flowers.

Genus	2020	2021	2022
*Andrena*	2	2	5
*Anthidium*		1	
*Anthophora*			8
*Apis mellifera*	2	46	18
*Ashmeadiella*	2	5	
*Atoposmia*			1
*Augochlorella*			1
*Centris*			1
*Colletes*		6	1
*Diadasia*	1	2	
*Epeolus*			1
*Eucera*			2
*Hesperapis*		2	
*Hoplitis*			1
*Hylaeus*		63	16
*Lasioglossum*	11	33	24
*Macrotera*	2		
*Megachile*	1	1	1
*Megandrena*			1
*Melissodes*			1
*Perdita*	168	21	169
*Stelis*		1	

**Table 3:** Locations of *A. californica* sites surveyed during 2020–2022. Sites are displayed as either being part of the Lake Mead National Recreation Area or part of Gold Butte National Monument.

Site Name	Code	GM GPS
Lake Mead National Recreation Area		
Apex	Apex	36.305500, −114.939444
Railroad	RR	36.314672, −114.947205
Bitter Spring 1	BS1	36.258327, −114.528040
Bitter Spring 2	BS2	36.287710, −114.522428
Bitter Spring 3	BS3	36.286681, −114.505937
Bitter Spring 4	BS4	36.288654, −114.499012
Steward Point	SP	36.386026, −114.413172
Pinto Ridge	PR	36.241706, −114.550132
Rainbow Garden North	RGN	36.176468, −114.961672
Rainbow Garden South	RGS	36.144443, −114.972828
Shooting Range	SR	36.135997, −114.981891
Pabco	Pb	36.205775, −114.912158
Ore Car Mine	OCM	36.212040, −114.700568
Borax Wash	BW	36.203463, −114.732925
**Gold Butte National Monument**		
Poppy City	PC	36.335103, −114.233219
Red Bluff North	RBN	36.456679, −114.251431
Red Bluff South	RBS	36.449705, −114.245201
Restoration Site	RS	36.504502, −114.195595
Black Butte	BB	36.499921, −114.201760
Helicopter Hill	HH	36.381543, −114.231045
Poppy Canyon	PCn	36.398943, −114.227713

## Data Availability

Data are presented in the manuscript. All specimens are housed in the U.S. National Pollinating Insects Collection, USDA-ARS Pollinating Insect Research Unit (PIRU), Logan, Utah.
